# LIF and let die: Generation, mechanistical, and structural characterization of bispecific antibodies triggering leukemia inhibitory factor (LIF) receptor activation independent of the cytokine LIF

**DOI:** 10.1016/j.jbc.2026.113634

**Published:** 2026-10-09

**Authors:** Julia Harwardt, Silke Pudewell, Kristina Keil, Britta Lipinski, Laura Unmuth, Thi Hong Hue Tran, Enrico Guarnera, Desislava Elter, Lukas Pekar, Doreen M. Floss, Jürgen Scheller, Andrew B. Ward, Monica L. Fernández-Quintero, Andreas Evers, Stefan Zielonka

**Affiliations:** 1 Antibody Discovery and Protein Engineering, NBE Technologies, Merck Healthcare KGaA, Darmstadt, Germany; 2 Institute of Biochemistry and Molecular Biology II, Medical Faculty and University Hospital Düsseldorf, Heinrich-Heine-University Düsseldorf, Düsseldorf, Germany; 3 Biomolecular Immunotherapy, Institute for Organic Chemistry and Biochemistry, Technical University of Darmstadt, Darmstadt, Germany; 4 Department of Integrative Structural and Computational Biology, The Scripps Research Institute, La Jolla, USA; 5 Novo Nordisk Foundation Initiative for Vaccines and Immunity, NIVI Research Center, Department of Immunology and Microbiology, University of Copenhagen, Copenhagen, Denmark

**Keywords:** antibody engineering, bispecific antibody, cytokine mimetic, IL-6-like cytokines, LIF, single domain antibody, surrogate cytokine, VHH, yeast surface display

## Abstract

Here, we report on the generation and characterization of single-domain antibody (sdAb)-derived bispecifics that mimic the function of LIF by triggering agonism on its heterodimeric receptor composed of the subunits LIF receptor (LIFR) and glycoprotein 130 (gp130). For this, LIFR-targeting sdAbs were identified by yeast surface display (YSD) after camelid immunization. Resulting 14 LIFR-specific paratopes were expressed in a bispecific antibody (bsAb) format, all harboring the identical gp130-addressing sdAb. Profiling of bsAbs on Ba/F3 cells expressing human LIFR as well as gp130 revealed LIF-like functionality of one bsAb as characterized by the induction of cell proliferation. Cryo-EM structural analysis revealed that the bsAb targets a distinct LIFR epitope, indicating the formation of a receptor architecture that differs from previously reported LIF signaling complex structures. In addition, the identified sdAb-based bispecific LIF mimetic elicited STAT3 phosphorylation in HeLa as well as TF-1 cells naturally expressing the LIF receptor subunits. Moreover, agonistic capabilities were further improved by antibody engineering. Taken together, we demonstrate that cytokine members of the IL-6 family can be mimicked by harnessing sdAbs as building blocks.

LIF is a pleiotropic cytokine, belonging to the IL-6 family of cytokines 1, 2, 3. As such, LIF exerts multiple, in part, opposing functions in various biological processes including hematopoiesis, immune regulation and embryonic development 4, 5. The cytokine is produced by many different cell types, for instance fibroblasts, macrophages, endothelial cells, adipocytes or chondrocytes and likewise, LIF acts on many different cells and tissues such as embryonic and hematopoietic stem cells, the immune cell compartment (*e.g.*, macrophages, T cells and B cells), chondrocytes, adipocytes as well as epithelial and endothelial cells 4, 5, 6. Originally identified for the ability to inhibit proliferation of certain leukemia cells (7), the multifaceted role of LIF in the physiological context has become evident within recent years (4). In this manner, LIF expression plays a crucial role for blastocyst implantation during pregnancy, where it is produced by the endometrial glands and acts on the endometrial epithelium enabling it to become receptive for blastocyst attachment 4, 8, 9, 10. Moreover, LIF is essential for maintaining the pluripotency of embryonic stem cells 5, 11. LIF, together with its receptor, also plays a major role in the development of the nervous system, where for example, it stimulates the maturation of astrocytes and oligodendrocytes 12, 13, 14, 15. Besides the nervous system, LIF maintains the homeostasis and regeneration of several other somatic tissues such as the intestine, muscle or the kidney (5). In addition, LIF acts on different immune cell subsets where it primarily exerts an immunosuppressive function. For example, LIF inhibits the differentiation and pro-inflammatory response in Th17 cells or dendritic cells and promotes monocyte differentiation into immunosuppressive macrophages *in vitro* 5, 16, 17, 18. Consequently, recombinant human (rh) LIF treatment emerged as promising strategy for immune-related diseases. On the other hand, multiple studies have shown that LIF harbors an oncogenic, tumor-promoting function as well as a key role in cachexia, as elegantly reviewed elsewhere 5, 19, 20, 21.

LIF signals through the heterodimeric receptor composed of LIFR as well as gp130 22, 23, 24. Hence, the trimeric complex of LIF:LIFR:gp130 results in downstream signaling involving cross-phosphorylation of constitutively associated Janus kinases (JAK), which subsequently serve as docking sites for STAT (signal transducer and activator of transcription) molecules (24). LIF is capable of binding independently to both of its receptor subunits, albeit with differences in affinities (KD_LIFR_ ∼ 1 nM vs. KD_gp130_ ∼ 80 nM) (22). Interestingly, also other IL-6-like family members share the LIFR:gp130 receptor components for signaling, such as oncostatin M (OSM) as well as cardiotrophin-1 (CT-1), ciliary neurotrophic growth factor (CNTF), and cardiotrophin-like cytokine (CLC), with some of those requiring an additional alpha chain receptor for signaling (3).

In the present work, we have generated and characterized bsAb-based mimetics for LIF. To this end, LIFR-specific sdAbs were identified from immunized camelids by harnessing YSD as platform technology (25). Subsequently, resulting paratopes were recombined with a single gp130-specific VHH (variable domain of the heavy chain of a heavy chain-only antibody) that was recently described as potent inhibitor of IL-6-type cytokine signalling, including LIF antagonism (26). Initial recombination yielding heterodimeric (1 + 1) bispecifics was achieved exploiting a process based on controlled Fab-arm exchange (cFAE) 27, 28, 29. Characterization of bsAbs on human LIFR as well as gp130-expressing Ba/F3 cells unveiled agonism of one bsAb candidate. Interestingly, this particular bsAb did not compete with (rh) LIF for binding to the LIFR subunit and hence addressed a different epitope on the receptor chain than the wild-type cytokine. Cryo-EM analysis subsequently confirmed that the bsAb specifically engages domain 2 (D2) of LIFR, presumably stabilizing a receptor conformation distinct from that induced upon natural ligand binding, while the anti-gp130 VHH targets an epitope overlapping with the LIF binding site on gp130. Eventually, we demonstrate that antibody engineering in terms of sdAb valencies and by modulating the spatial orientation of individual paratopes within the overall molecular design enables augmented LIF-like agonism. Essentially, this study provides mechanistic evidence that cytokine-like agonism can be induced on receptors belonging to the IL-6 cytokine family by exploiting sdAb-derived bispecifics which target and cross-link the respective individual receptor subunits. To our knowledge, this work also presents the first cryo-EM structure of a VHH-based bispecific antibody simultaneously engaging both subunits of a heterodimeric cytokine receptor, revealing the architecture of the trimeric bsAb–receptor complex and providing structural insight into antibody-mediated cytokine receptor agonism.

## Results

### Isolation of LIFR-specific VHH building blocks by camelid immunization and YSD

To isolate VHHs targeting the LIFR, a llama (*lama glama*), an alpaca (*vicugna pacos*) and a huarizo (*llama glama x vicugna pacos*) were immunized four times over a total period of 43 days using the extracellular domain (ECD) of LIFR protein. Based on the RNA isolated from the PBMC repertoire and subsequent cDNA synthesis, a YSD library with an estimated library size of 3.1 × 10^8^ unique clones was generated. A two-dimensional sorting strategy was applied to screen for full-length VHH display (*via* hemagglutinin (HA) tag staining) and binding functionality (*via* histidine (His) tag staining) by fluorescence activated cell sorting (FACS) (Fig. 1*A*). Utilizing an antigen concentration of 1 μM, a LIFR binding population was successfully enriched after two rounds of sorting (Fig. 1*B*). Of the 192 clones derived from the enriched population that were sent for sequencing, 172 unique clones were identified that exhibited high sequence diversity, particularly in the Complementarity-determining regions (CDRs) (Fig. 1*C*). A clustering strategy was applied based on the diversity within CDR3 to nominate 28 VHHs for reformatting, which were subsequently expressed in Expi293 cells and purified *via* protein A chromatography.

**Figure 1:**
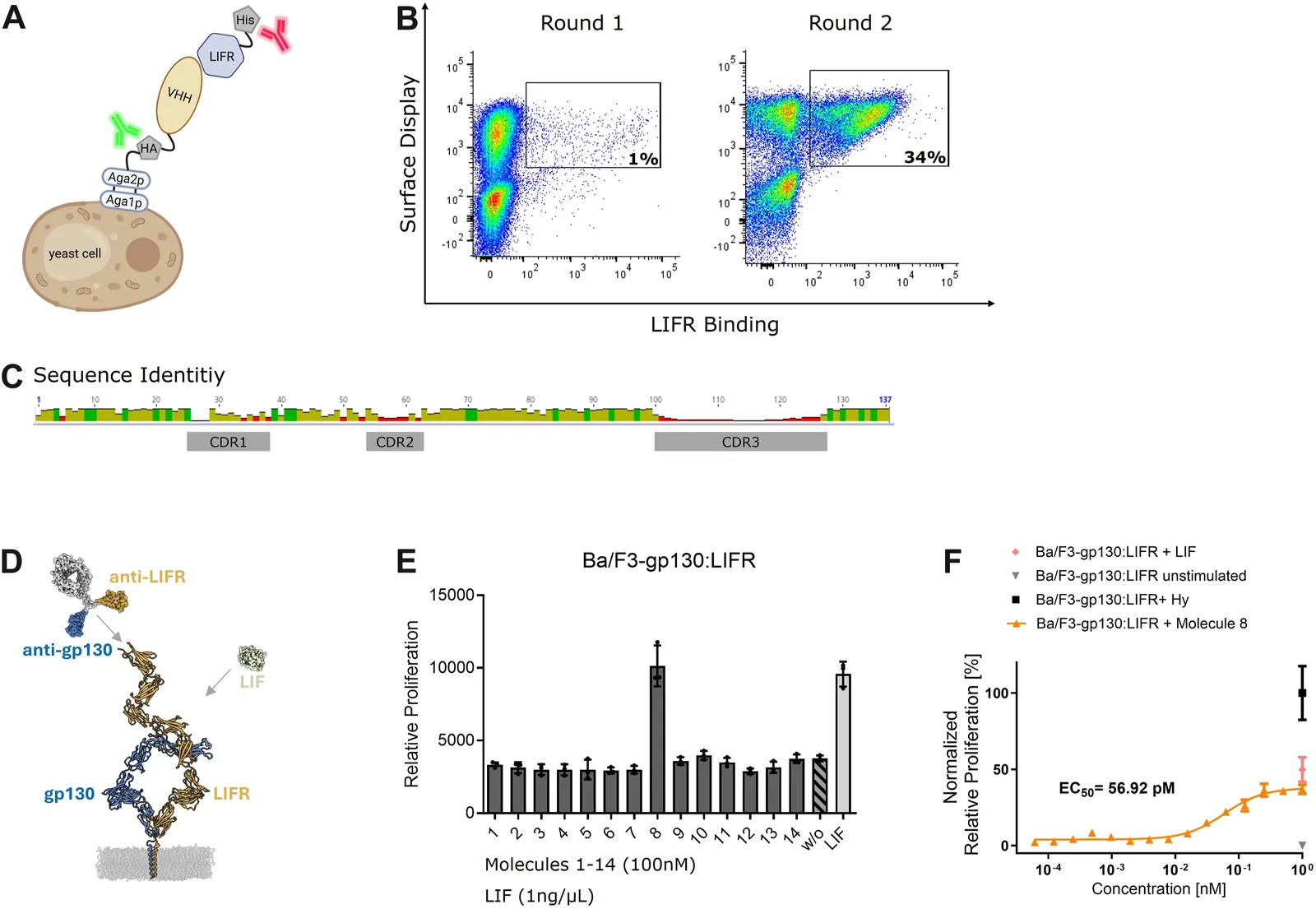
**YSD-based strategy for the generation of LIF mimetics based on sdAb-derived bispecifics.***A*, schematic illustration of yeast surface display based on *S. cerevisiae* strain EBY100. Fluorophore-labeled detection reagents allow for FACS-based gating of VHH display (HA tag) and LIFR binding (His tag). Generated using www.biorender.com. *B*, camelid immunization followed by YSD results in the enrichment of LIFR specific sdAbs. In the first and second round of selection, 1 μM (rh) LIFR was utilized. A two-dimensional sorting strategy was applied to select for full-length VHH display in addition to antigen binding. Percentage of cells in sorting gates are shown. *C*, graphical sequence alignment of 172 unique sdAb clones addressing LIFR originating from YSD library sorting. CDRs are highlighted. *Red bars* represent high sequence diversity at amino acid level and *green bars* indicate high sequence conservation. Alignment conducted with MUSCLE alignment tool using Geneious Prime 2025.0.2. *D*, Bispecific sdAb binding to the heterodimeric LIF receptor by engaging LIFR (*orange*) and gp130 (*blue*) and thus eliciting downstream signaling results in the proliferation of Ba/F3-gp130:LIFR cells. Structural visualization was generated with ChimeraX (64). *E*, Proliferation assay of Ba/F3-gp130:LIFR cells stimulated with 1 ng/μl (rh) LIF or 100 nM of bispecific molecules 1 to 14. The negative control (w/o) represents unstimulated cells. The values are measured in triplicates. *F*, proliferation assay of Ba/F3-gp130:LIFR cells. Molecule 8 was titrated to the cells in a concentration range between 0.061 pM and 1 nM. Hyper-IL-6 (Hy) was used as positive control. Every value is measured in triplicates and all assays were performed in three independent experiments (n = 3).

### A LIFR and gp130-specific sdAb-derived bispecific triggers robust LIF-like receptor agonism

Out of the 28 VHH-Fc proteins evaluated, 14 molecules exhibited specific binding to the recombinant human LIFR protein, as evaluated by biolayer interferometry (BLI) (Fig. S1*A*). In this regard, affinities of LIFR-specific molecules were in the single-digit to double-digit nanomolar range. Those were nominated for bsAb creation harnessing a gp130-targeting VHH that has been recently described (26). This particular gp130-specific sdAb was previously utilized as an inhibitor of IL-6-type cytokine signaling by interfering with the gp130 binding site of IL-6, IL-11, LIF and other IL-6-type cytokines. We set out to scrutinize whether this specific antagonist could be converted into an agonist by combining it with one of the VHHs targeting the other chain of the LIF receptor complex. To combine the LIFR-specific sdAbs with the gp130-specific VHH, we exploited the cFAE technology 27, 28, 29. This technique leverages IgG4-like single-point mutations in the IgG1 CH3 domain, which destabilizes the IgG homodimer and promotes heterodimer formation under reducing conditions. Specifically, the F405L mutation was introduced in the Fc region of the gp130-specific sdAb, while the corresponding K409R mutation was incorporated in the LIFR-specific sdAbs. Additionally, the Fc region was effector-silenced to exclude Fc-mediated effector functions.

The resulting bsAbs were analyzed regarding their agonistic properties using Ba/F3 cells that express LIFR and gp130 (Ba/F3-gp130:LIFR), allowing for proliferation in the presence of LIF (Fig. 1, *D*–*F*). One of the molecules (#8) was identified as an agonist and demonstrated a proliferative effect on the Ba/F3-gp130:LIFR cells with potencies (EC_50_ of proliferation induction) in the double-digit pM range, comparable to the range observed for the cytokine LIF (Figs. 1, *E*, *F* and S2). Notably, BLI-based binding competition experiments demonstrated that molecule #4 targets an overlapping epitope - or a site in close proximity - on LIFR relative to the identified agonist #8, despite the absence of agonistic activity (Figs. 1*E* and 2*A*). However, the CDR3 region of the LIFR-directed paratope of molecule #4 differed substantially from that of the LIFR-targeting VHH #8 (Fig. S1*B*). Consequently, it is tempting to speculate that it is really the fine epitope that is being addressed by a certain VHH that discriminates between agonism and non-agonism. The remaining 12 LIFR binders target different epitopes than that bound by agonist #8 (Fig. 2*A*). Beyond, the sequential binding of agonist #8 and the natural ligand (rh) LIF to the target LIFR demonstrated that both #8 and LIF are able to bind to LIFR simultaneously, giving evidence that they recognize distinct epitopes on the receptor subunit (Fig. 2*B*). This was also true for all other 13 LIFR binders that did not exhibit agonistic activity (Fig. S3).

**Figure 2:**
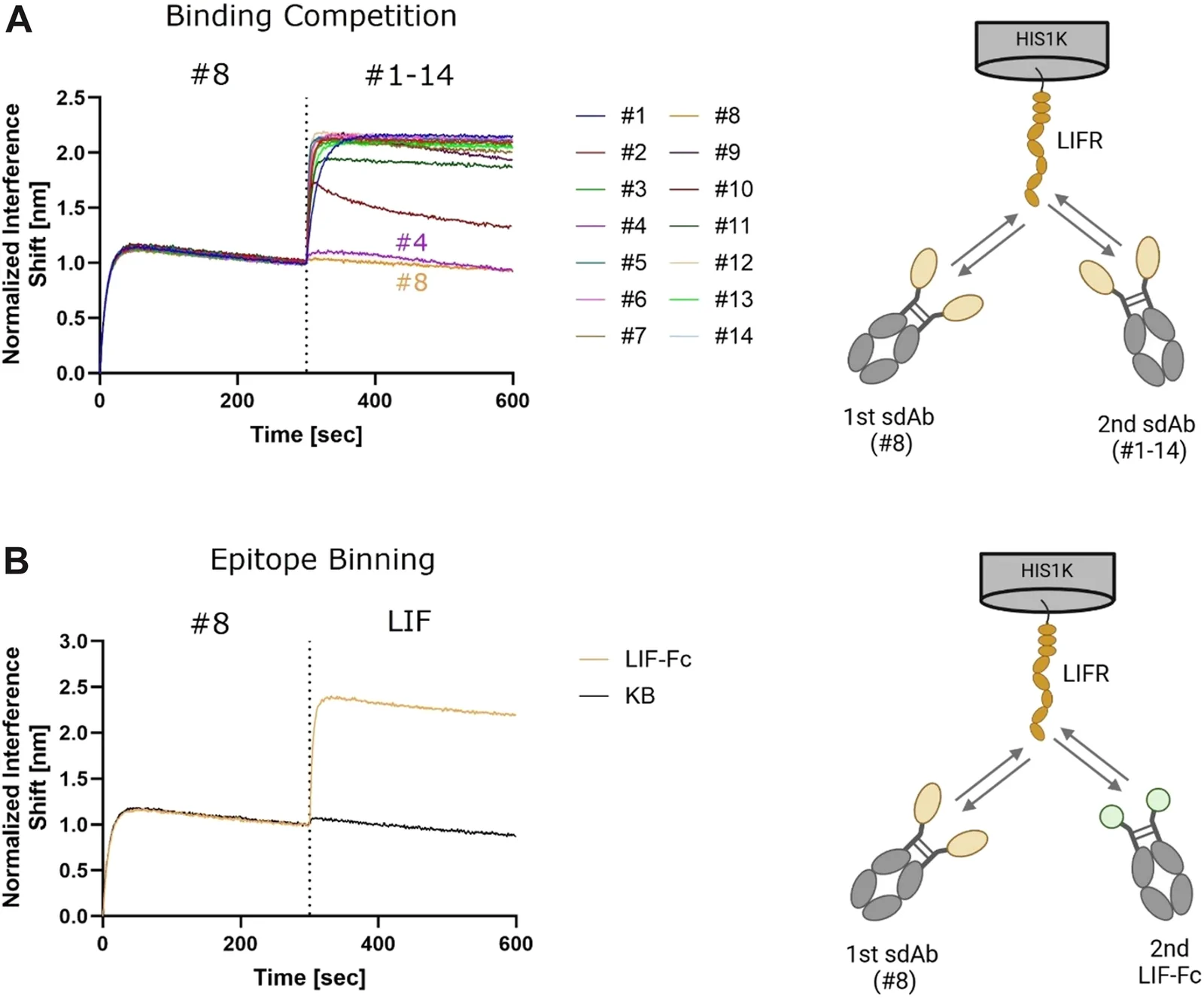
**Binding characterization of identified LIFR agonist *via* Biolayer interferometry measurements.***A*, BLI-assisted binding competition assay. LIFR was loaded onto HIS1K biosensors followed by a first association of molecule #8 for 300 s and a second association of molecules #1 to 14 for 300 s. *B*, BLI-assisted epitope binning assay. LIFR was loaded onto HIS1K biosensors followed by a first association of molecule #8 for 300 s and a second association of LIF-Fc for 300 s.

### Cryo-EM and molecular modeling studies rationalize agonism capacities of engineered LIF mimetics

As outlined in the introduction, upon LIF engagement, the receptor subunits undergo heterodimerization to form the ternary LIF:LIFR:gp130 signaling complex (Fig. 3, *A* and *B*), which positions the constitutively associated JAK family kinases (primarily JAK1, JAK2, and TYK2) for trans-phosphorylation 24, 30. This triggers further downstream signaling, involving predominantly STAT3 phosphorylation as well as PI3K/AKT and MAPK pathways 15, 31. Structural studies have revealed that the spatial geometry of the LIFR:gp130 heterodimer (Fig. 3*B*) is critical for efficient signaling 24, 32. The receptor complex forms an asymmetric arrangement where the extracellular and transmembrane segments join as a continuous, rigid unit, optimally positioning the intracellular JAK kinases for activation. Consequently, as illustrated in Figure 3*C*, successful LIF mimetics must not only bind both receptor subunits but also orient them in a signaling-competent geometry.

**Figure 3:**
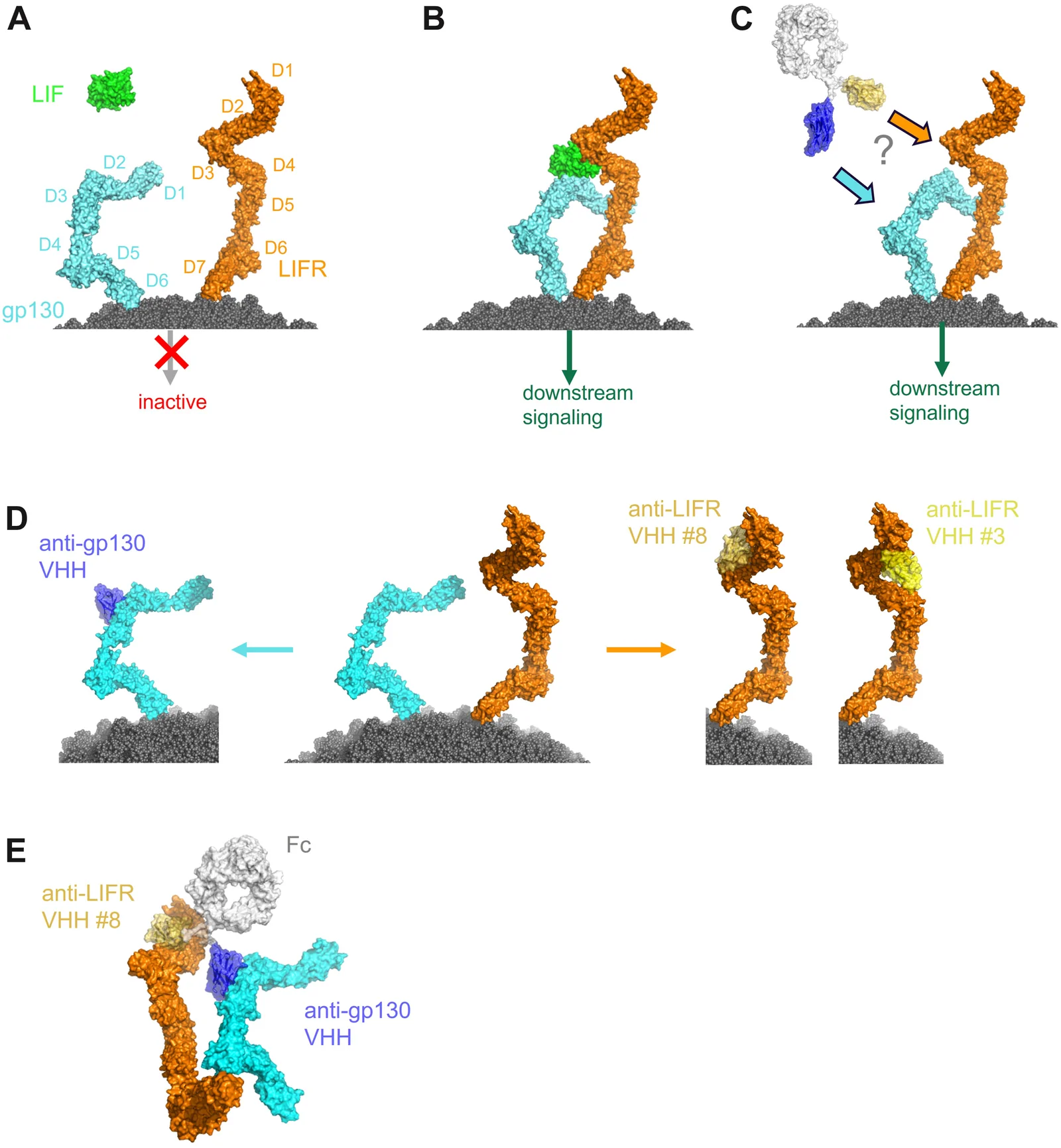
**Structural basis for bispecific antibody-mediated LIF receptor agonism.***A*, in the absence of LIF, gp130 (*cyan*) and LIFR (*orange*) exist as separate, inactive receptor subunits at the cell surface that do not productively signal. Domains of gp130 (D1–D6) and LIFR (D1–D7) are labeled. *B*, upon LIF (*green*) engagement, gp130 and LIFR undergo heterodimerization to form the ternary LIF:LIFR:gp130 signaling complex, triggering downstream signaling (pdb code 8v29). *C*, schematic illustration of the LIF mimicry concept: a bispecific antibody comprising an anti-gp130 VHH (*blue*) and an anti-LIFR VHH (*orange*-*yellow*) is hypothesized to simultaneously engage both receptor subunits, potentially inducing a signaling-competent receptor geometry. *D*, individual binding modes of the anti-gp130 VHH (*blue*, *left panel*), the anti-LIFR VHH #8 (*orange*-*yellow*, *center*-*right panel*) and the anti-LIFR VHH #3 (*light*-*yellow*, *right panel*) to their respective receptor subunits gp130 (*cyan*) and LIFR (*orange*), as generated by density-informed structural models derived from cryo-EM data (Fig. 4). *E*, density-informed structural model of the trimeric bsAb–receptor complex, showing simultaneous engagement of gp130 (*cyan*) and LIFR (*orange*) by the bispecific antibody comprising the anti-gp130 VHH (*blue*) and the anti-LIFR VHH #8 (*yellow*/tan), bridging both receptor subunits. Notably, the relative orientation of LIFR and gp130 within the bsAb-induced complex is distinct from the receptor arrangement observed in the native LIF:LIFR:gp130 signaling complex (*B*), indicating that the bispecific antibody stabilizes an alternative receptor assembly. Structures and models were visualized using PyMOL.

To elucidate the structural basis responsible for the agonistic activity of VHH #8, we performed cryo-electron microscopy (cryo-EM) on LIFR in complex with VHH #8, which is agonistic in combination with the anti-gp130 VHH, and a non-agonistic VHH (#3). Already at the level of the 2D class averages, it became apparent that the two VHHs recognize distinct epitopes on LIFR (Fig. 4, *A* and *B*). Subsequent 3D reconstructions revealed that VHH #8 targets the D2 domain of LIFR, whereas VHH #3 binds at the intersection between the D1 and D2 domains (Fig. 5). The agonistic activity of VHH #8 within the bsAb is likely driven by its shifted epitope and altered interaction surface, potentially stabilizing a specific signaling-competent receptor conformation.

**Figure 4:**
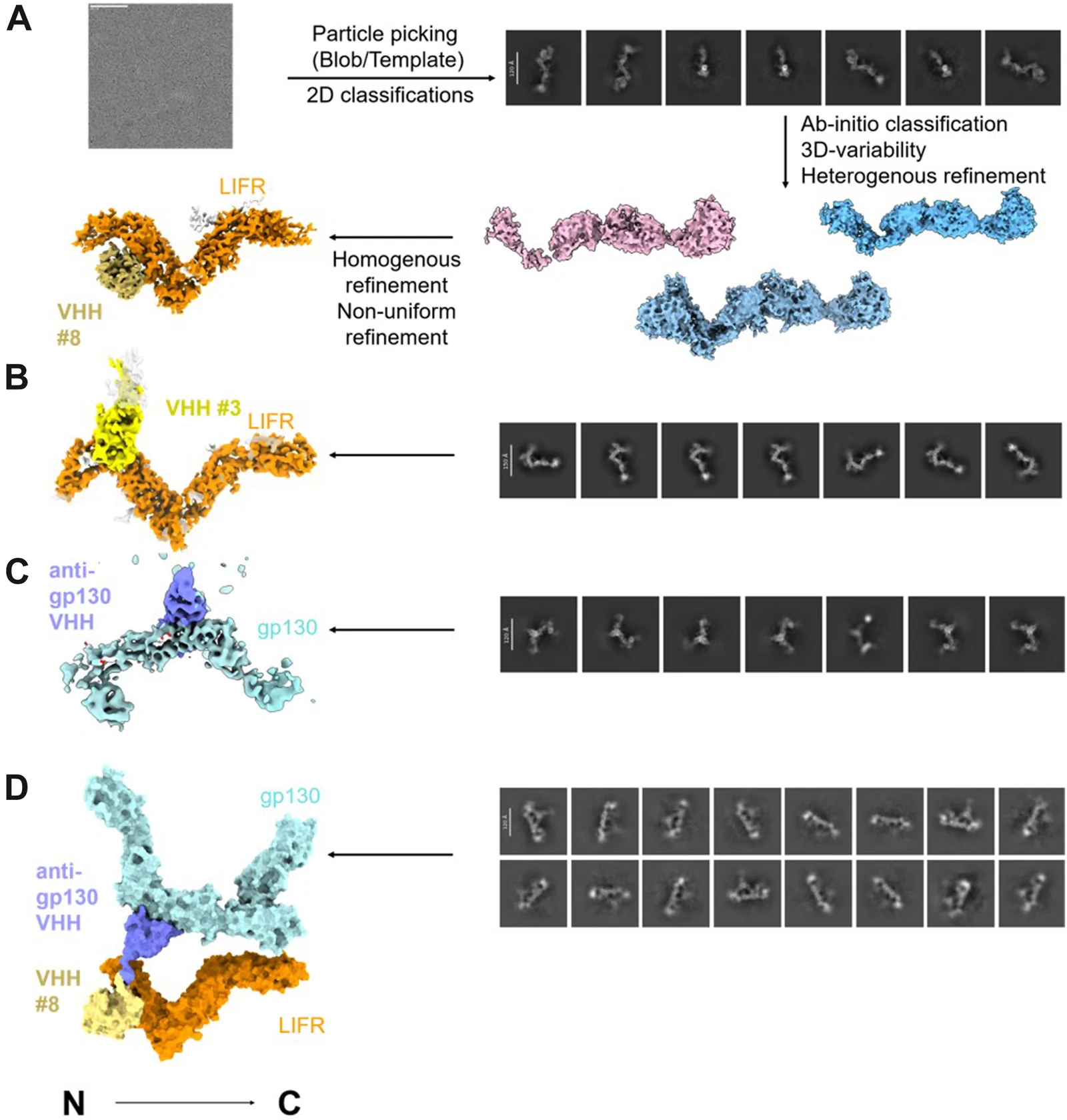
**Cryo-EM data collection, processing workflow, and structural characterization of VHH–receptor complexes.***A*, Cryo-EM processing pipeline for the LIFR:VHH #8 complex. A representative micrograph (*top left*) was used for particle picking employing blob and template-based approaches, followed by 2D classification (*top right*). Representative 2D class averages are shown. Ab initio classification, 3D variability analysis and heterogeneous refinement yielded distinct 3D classes (*center*), which were subsequently subjected to homogeneous and non-uniform refinement, resulting in the final cryo-EM density map of the LIFR (*orange*):VHH #8 (*orange*-*yellow*) complex (*left*). *B*, final cryo-EM density map (*left*) and representative 2D class averages (*right*) of LIFR (*orange*) in complex with the non-agonistic VHH #3 (*yellow*), revealing binding at the intersection of the D1 and D2 domains, distinct from the epitope targeted by VHH #8. *C*, final cryo-EM density map (*left*) and representative 2D class averages (*right*) of gp130 (*cyan*) in complex with the anti-gp130 VHH (*blue*/*purple*). *D*, representative 2D class averages (*right*) and density-informed structural model (*left*) of the parental bispecific antibody in complex with both LIFR (*orange*) and gp130 (*cyan*), with the anti-LIFR VHH #8 (*orange*-*yellow*) and the anti-gp130 VHH (*blue*/*purple*) simultaneously engaging their respective receptor subunits. The N→C arrow at the *bottom* indicates the N- to C-terminal orientation of the receptor extracellular domains shown in *panels A-D*. Scale bars, 100 Å. Structural visualizations were generated using UCSF ChimeraX.

**Figure 5:**
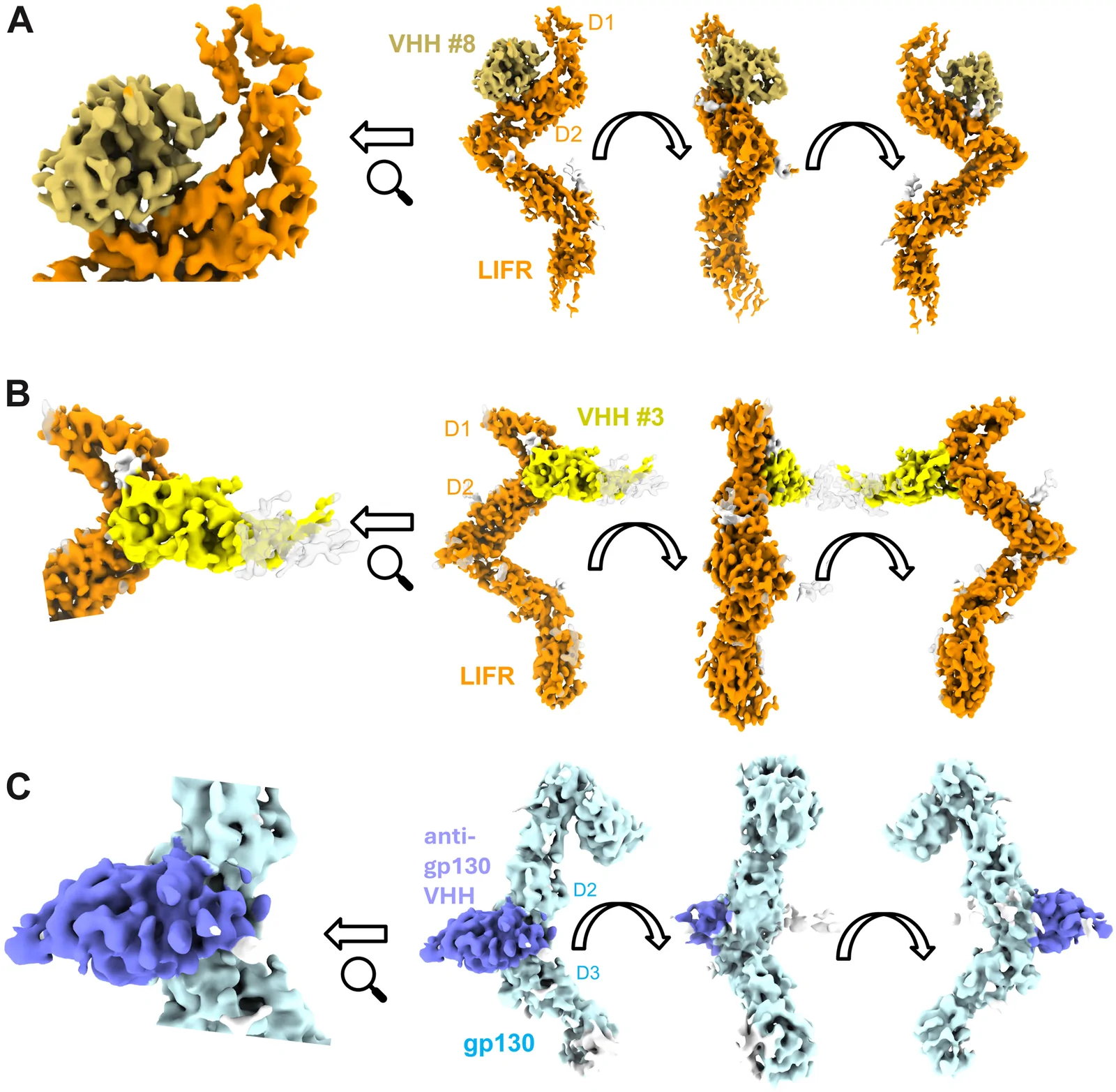
**Enlarged and rotated views of cryo-EM density maps of VHH-receptor complexes.** Close-up cryo-EM density maps are shown on the *left*, and rotated views of the same complexes are shown on the *right*. *A*, agonistic VHH #8 (*orange*-*yellow*) engages LIFR domain D2. *B*, non-agonistic VHH #3 (*yellow*) binds at the interface between LIFR domains D1 and D2. *C*, the anti-gp130 VHH (*blue*/*purple*) engages gp130 domain D3. Receptor domains contacted by the VHHs are labeled. N-terminal regions are oriented toward the *top* of the close-up panels and C-terminal/membrane-proximal regions toward the *bottom*. *Curved arrows* indicate rotation of the same density maps. All maps were visualized and rendered in UCSF ChimeraX.

Due to pronounced flexibility of the VHH-receptor complexes, the obtained cryo-EM maps (Fig. 4, *A* and *B*) did not reach sufficient resolution (∼4–5 Å) for confident atomic model building. Enlarged and rotated views of the final cryo-EM density maps are provided in Figure 5; the receptor domains contacted by the VHHs (LIFR D2; LIFR D1/D2 junction; gp130 D3) are labeled. Consequently, the resulting density-informed models are not deposited in the PDB; the EMDB-deposited maps constitute the primary structural data of this study. Nevertheless, the experimental density information was integrated with structure prediction approaches to generate density-informed structural models and interpret the binding modes of the VHHs. Figure 3*D* shows VHH #8 (yellow-orange) and VHH #3 (yellow) in complex with LIFR (orange), highlighting the distinct epitopes targeted by the two VHHs. The cryo-EM density and the derived density-guided structural model of the gp130:anti-gp130 VHH complex confirm that the VHH engages an epitope overlapping with the LIF binding site on gp130 (see Fig. 3*B* *versus* 3*D* and 4*C*).

In addition to the individual VHHs, we also aimed to obtain a cryo-EM structure of the resulting bsAb, designed to bridge gp130 and LIFR. Although we could not obtain a high-resolution reconstruction of the fully assembled receptor complex, the 2D class averages suggest the formation of a distinct receptor architecture (Figs. 3*E* and 4*D*) compared to the previously reported LIF signaling complex structures (PDB accession code: 8v29). These observations support the proposed bridging mechanism of the bsAb and indicate the formation of alternative receptor assemblies that may contribute to its functional activity.

Interestingly, neither AlphaFold2, AlphaFold3, or Boltz-2 accurately predicted the experimentally observed binding interfaces identified by the cryo-EM maps, highlighting the challenges current structure prediction approaches face in modeling flexible receptor-VHH interactions of previously uncharacterized epitopes.

### Antibody engineering facilitates the generation of sdAb-derived LIF mimetics with augmented agonism capacities

To enhance the functional properties of the herein generated LIF mimetic, antibody architecture engineering was applied, investigating the influence of paratope valencies and spatial orientation. The different antibody designs are shown in Figure 6*A*. Besides the monovalent (1 + 1; parental) VHH-Fc format of the parental molecule, several additional molecular design architectures were explored. The first design relies on tandem VHH arrangements that were engrafted onto the hinge region of an effector-silenced IgG1 Fc domain (2 + 2; tandem). The anti-LIFR VHH #8 and the anti-gp130 VHH were separated by a five-amino-acid Gly4Ser linker. Both orientations, anti-LIFR VHH (at the *N*-terminus) followed by anti-gp130 VHH (tandem_LIFRxgp130_) and *vice versa* (tandem_gp130xLIFR_) were assessed. Secondly, in a format referred to as ‘hidden IgG’ (hiIgG) the VH and VL of a conventional antibody were replaced by two individual VHH domains (33). One VHH was engrafted onto the CH1 domain of the effector-silenced IgG1 heavy chain and the other VHH was engrafted onto the constant region of the lambda light chain, resulting in an IgG-like molecule that is bispecific by design and allows for bivalent targeting of both antigens (bispecific, tetravalent). Also here, both orientations, anti-LIFR VHH engrafted onto the heavy chain and anti-gp130 VHH onto the light chain (hiIgG_LIFRxgp130_) and *vice versa* (hiIgG_gp130xLIFR_) were evaluated. Third, one-armed (oa) variants of the bispecific, tetravalent antibodies were designed (referred to as oa tandem and oa hiIgG, respectively), resulting in bispecific bivalent antibodies. To ensure heavy chain heterodimerization, the strand-exchange engineered domain (SEED) technology, relying on beta-strand exchanges of IgG and IgA CH3 constant domains, was employed for all relevant constructs, including the parental (1 + 1) heterodimeric bispecific (34). Of note, all constructs were produced as Fc effector-silenced derivatives.

**Figure 6:**
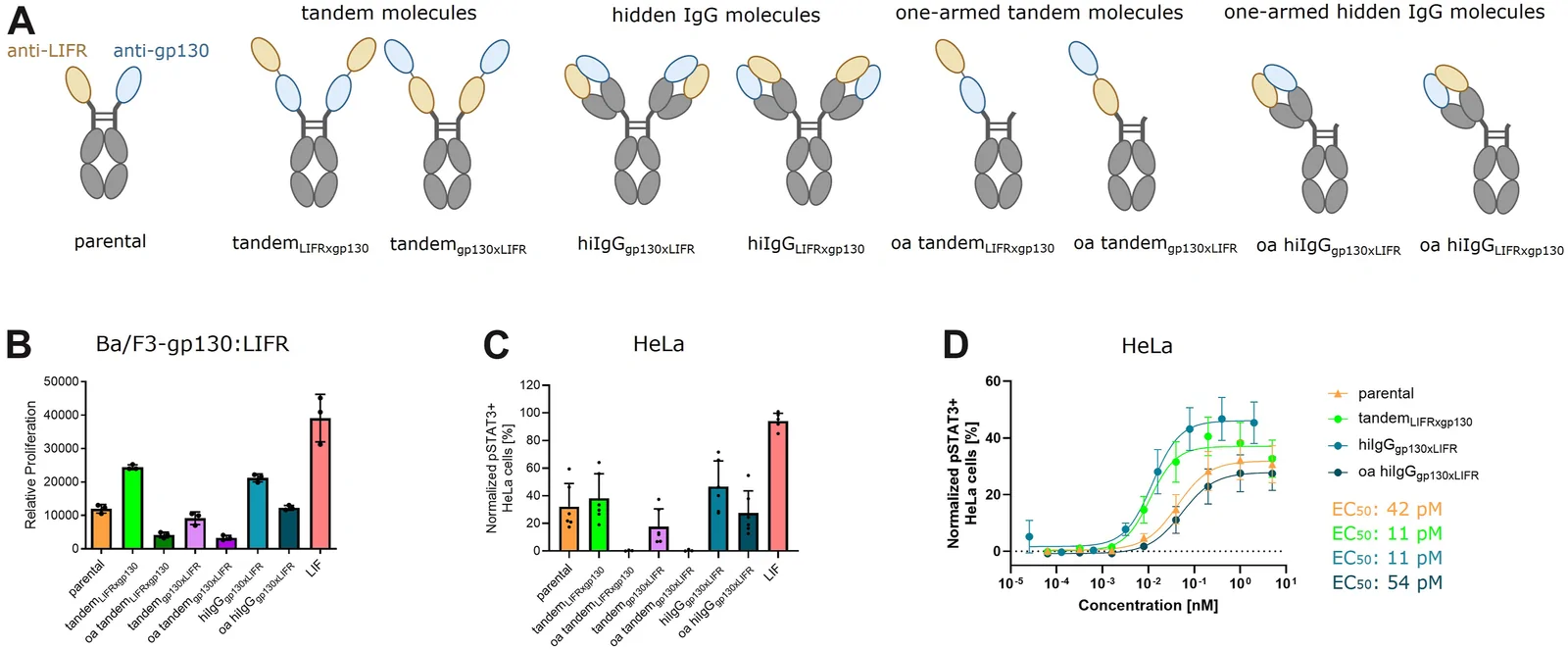
**Valency engineering further enhances the LIF-like agonism.***A*, two different bsAb architectures consisting of different valencies and orientations of the LIFR targeting VHH (*orange*) and the gp130 targeting VHH (*blue*) were functionally compared. The first design is based on tandem VHH arrangements, while in the hidden IgG format, one VHH was engrafted onto the constant region of the heavy and light chain, respectively. *B*, proliferation assay of Ba/F3-gp130:LIFR cells stimulated with 1 ng/μl LIF or 1 nM of the engineered molecules. The negative control (w/o) represents unstimulated cells. The values are measured in triplicates. *C and D*, intracellular staining of pSTAT3^+^ HeLa cells after 40 min incubation with LIF or LIFR agonists at different concentrations. Mean values ± SD of 6 independent measurements are depicted in the bar graph. The dose–response curves show mean values ± SEM and were fitted using a non-linear four parameter fit in GraphPad Prism.

Production of the different bsAb architectures was conducted in Expi293 cells, followed by protein A purification. As a cut-off in terms of purities, we considered target peaks in analytical size exclusion chromatography of more than 80% as sufficient for further characterization. As shown in Table 1 and Figure S4, most of the constructs exhibited high purities post protein A purification. However, hiIgG having the VHH targeting LIFR engrafted onto the heavy chain as well as the paratope directed against gp130 grafted on the light chain (hiIgG_LIFRxgp130_) only displayed purities of 39.1%. The corresponding one-armed derivative (oa hiIgG_LIFRxgp130_) also showed a low target peak of 62.5%. Consequently, both molecules were excluded from further characterization.

**Table 1:** Biophysical and Functional Properties of the Generated Bispecific LIF Agonists

Sample	Valency	SEC purity after protein A (%)	EC_50_ proliferation of Ba/F3-gp130:LIFR cells (pM)	EC_50_ pSTAT3 activation of HeLa cells (pM)	EC_50_ pSTAT3 activation of TF-1 cells (pM)
parental	1 + 1	96.6	57	42	39
tandem_LIFRxgp130_	2 + 2	93.5	27	11	9
oa tandem_LIFRxgp130_	1 + 1	95.1	-	-	-
tandem_gp130xLIFR_	2 + 2	94.0	-	-	-
oa tandem_gp130xLIFR_	1 + 1	97.2	-	-	-
hiIgG_gp130xLIFR_	2 + 2	83.5	18	11	12
oa hiIgG_gp130xLIFR_	1 + 1	94.0	58	54	49
hiIgG_LIFRxgp130_	2 + 2	39.1	-	-	-
oa hiIgG_LIFRxgp130_	1 + 1	62.5	-	-	-

All other engineered constructs were assessed for their capability to induce proliferation in Ba/F3-gp130:LIFR cells at a fixed concentration of 1 nM (Fig. 6*B*). In this respect, engineered one-armed molecules did not induce appreciably stronger proliferation of Ba/F3-gp130:LIFR cells when compared with the parental bsAb. BLI binding experiments demonstrated that all bispecific constructs are capable of binding LIFR and gp130 simultaneously (Fig. S5). In fact, the one-armed tandem molecules in both orientations did not trigger any meaningful proliferation at all, while the one-armed hidden IgG molecule (oa hiIgG_gp130xLIFR_) was comparable to the parental molecule. Interestingly, tetravalent bispecific molecule tandem_gp130xLIFR_ was weaker in eliciting cell proliferation at this fixed concentration compared to the parental molecule, while the corresponding format in the other orientation, tandem_LIFRxgp130_, induced significantly stronger proliferation. Of note, for tandem_LIFRxgp130_ we witnessed stronger binding capacities against (rh) LIFR by BLI, demonstrated by higher interference pattern shifts at identical concentrations than for tandem_gp130xLIFR_ (Fig. S6*A*). For (rh) gp130 binding it was the other way around, where tandem_LIFRxgp130_ showed slightly reduced binding capacities compared to tandem_gp130xLIFR_ (Fig. S6*B*). Within the overall architecture of tandem_LIFRxgp130_ the VHH targeting LIFR is located at the *N*-terminus and thus enables uncompromised binding, whereas reduced binding capacities for the inner paratope suggest steric hindrance. Hence, this is giving some evidence that for this particular paratope combination, unaffected binding to the LIFR subunit seems to be of utmost importance to induce proliferation of the LIF receptor complex displaying Ba/F3 cells. Since the corresponding one-armed molecule (oa tandem_LIFRxgp130_) did not induce meaningful proliferation, it can be suggested that simultaneous LIFR and gp130 binding of the tetravalent molecule tandem_LIFRxgp130_ occurs across opposing antibody arms, thereby forming two receptor complexes, consistent with the observed increase in potency. Importantly, also hidden IgG derivative hiIgG_gp130xLIFR_ induced stronger proliferation in these cells at this single concentration when compared with the parental molecule, clearly indicating that valency engineering enables a stronger LIF-like agonism.

For all the engineered derivatives that were at least comparable to the parental format, we investigated induced proliferation of Ba/F3-gp130:LIFR cells in a dose titration (Fig. S7). While potencies were in the same range for the parental molecule (Fig. 1*F*) and for all engineered constructs tested (double-digit picomolar range), we observed differences in maximum proliferation compared to the cytokine (rh) LIF. Here, the bivalent bispecific format oa hiIgG_gp130xLIFR_ was clearly compromised when compared with the bispecific tetravalent molecules, confirming that the LIF-like agonism is enhanced by valency engineering.

### Engineered LIF mimetics facilitate STAT3 phosphorylation in cell lines naturally expressing the LIF receptor (LIFR:gp130)

Next, we wanted to assess whether induced proliferation in Ba/F3 cells stably expressing the LIF receptor also translates into agonism on cell lines naturally expressing both LIF receptor components. It is known that HeLa cells express the LIF receptor and hence are responsive to the cytokine LIF 35, 36. To quantify agonism by the generated LIF mimetics, we specifically looked at intracellular phosphorylation of STAT3 after 40 min of sample incubation at 1 nM using flow cytometry. Also in this assay system, the one-armed tandem molecules in both orientations did not trigger any meaningful STAT3 phosphorylation at all, while the one-armed hidden IgG molecule (oa hiIgG_gp130xLIFR_) was comparable to the parental molecule (Fig. 6*C*). Consistent with BLI affinity measurements and Ba/F3-gp130:LIFR cell proliferation experiments, tandem_gp130xLIFR_ demonstrated a decreased pSTAT3 signal compared to tandem_LIFRxgp130_. Molecule hiIgG_gp130xLIFR_ revealed a higher potency and efficacy than its one-armed variant, demonstrating the impact of additional valencies (Fig. 6*D*). Overall, the STAT3 phosphorylation data confirms that the orientation and number of paratopes have a significant impact on the biologic function. While the addition of two VHHs to the one-armed tandem_LIFRxgp130_ molecule (resulting in tandem_LIFRxgp130_) influences the overall agonistic properties of the molecule, valency engineering also further enhances LIF-like agonism in the hidden IgG format. Both bispecific tetravalent engineered LIF mimetics (tandem_LIFxgp130_ and hiIgG_gp130xLIFR_) exhibited comparable agonism capacities. Comparable results were obtained in pSTAT3 measurements on TF-1 cells naturally expressing LIFR and gp130 (37) (Fig. S8).

## Discussion

Cytokines are potent immune mediators, making them attractive as therapeutics for a variety of indications. However, their short blood half-lives, manufacturing complexities and serve side effects caused by low specificity and dose-limiting toxicities limit their clinical use (38). Numerous clinical trials are currently investigating cytokines, including engineered or modified cytokine versions to overcome the inherent challenges 39, 40, 41.

The pleiotropic cytokine LIF is involved in various biological processes including hematopoiesis, immune regulation and embryonic development, making it a promising target for new therapeutic approaches 4, 5. It’s role in reproduction, fertility and embryonic implantation is addressed in clinical trials (42). Additionally, LIF is described as an interesting target for autoimmunity. By inducing the differentiation of Tregs and inhibiting the proinflammatory response in Th17 cells, DCs and macrophages, LIF exerts an immunosuppressive effect. Furthermore, recombinant LIF in a nanoparticle formulation has been reported to cross the blood-brain barrier and retard the progression of multiple sclerosis (5). In general, the therapeutic use of recombinant cytokines can elicit anti-drug antibodies (ADAs) which may also cross-react with the endogenous cytokine, potentially neutralizing its biological function (43). This risk is minimized using antibody-based cytokine mimetics that do not share sequence identity with endogenous cytokines.

The herein presented study provides an alternative route for harnessing LIF functionalities and is giving mechanistic evidence that antibody engineering facilitates the generation of LIF mimetics with tailored biological functionalities. We generated a sdAb-based bispecific molecule that mimics LIF biology by cross-linking LIFR and gp130. For this, specific VHHs were isolated using yeast surface display following camelid immunization. To cross-link LIFR and gp130, a gp130-targeting VHH, described to interfere with the gp130 binding site of LIF, was combined with the isolated LIFR-targeting sdAbs. One bispecific molecule demonstrated LIF-like agonism converting the gp130-targeting antagonist into an agonist when being combined with this specific LIFR-targeting VHH. The cytokine LIF targets both receptor subunits with different affinities, with the LIFR subunit addressed with higher affinity (KD LIFR = 1 nM; KD gp130 = 80 nM) (22). Interestingly, the identified bispecific parental LIF mimetic (1 + 1 architecture) exhibits a reversed binding affinity profile (KD LIFR = 130 nM; KD gp130 = 1 nM). Similarly, all engineered antibody variants target LIFR with lower affinity than gp130. While the gp130-targeting VHH interferes with the LIF binding site, the LIFR-targeting VHH addresses a different epitope than the cytokine LIF. Cryo-EM measurements confirmed that the LIFR-targeting VHH #8 specifically engages LIFR D2, suggesting the stabilization of a unique receptor architecture. Although a high-resolution reconstruction of the fully assembled bsAb:LIFR:gp130 complex could not be obtained, the 2D class averages of the bsAb in complex with both receptor subunits indicate the formation of a receptor arrangement that is geometrically distinct from that observed in the native LIF:LIFR:gp130 signaling complex. This is particularly noteworthy, as it demonstrates that productive downstream signaling, as evidenced by STAT3 phosphorylation and Ba/F3 cell proliferation, can be achieved even when the receptor subunits are brought into an orientation that deviates from the geometry induced by the natural cytokine. This finding is reminiscent of observations made for other engineered receptor agonists, where alternative receptor geometries were shown to be compatible with, or even required for, efficient signaling 44, 45. It further supports the notion that the LIFR:gp130 heterodimer tolerates a degree of geometric flexibility in its extracellular arrangement while still enabling productive intracellular JAK-STAT signal transduction. It should be noted that, in common with the published cryo-EM structures of the LIF:LIFR:gp130 (PDB: 8D6A) and OSM:LIFR:gp130 (PDB: 8V29) assemblies, our structural analysis was performed on soluble extracellular domains in the absence of a lipid bilayer. While this represents the accepted standard in the field, we acknowledge that membrane embedding may impose additional geometric constraints on receptor subunit orientation that are not fully captured in a soluble ECD system, and the receptor arrangement in a native membrane context may therefore differ from what is observed here. Furthermore, a key distinction between antibody-mediated and cytokine-mediated receptor engagement is the inherent flexibility of the IgG hinge region. While LIF forms a rigid, stoichiometrically defined ternary complex, the hinge of the bispecific antibody introduces rotational and translational degrees of freedom between the two receptor-binding arms, potentially allowing accommodation of a broader range of receptor orientations. We therefore present the cryo-EM data as structural evidence consistent with an alternative extracellular receptor geometry, rather than as a definitive visualization of the signaling-competent membrane state. Future studies in membrane-mimetic systems, such as nanodisc-reconstituted receptors, will be needed to fully address this question.

Another isolated LIFR-targeting VHH (#4) that addresses a similar epitope as the identified agonist (#8) did not show agonistic activity when combined with the same gp130-specific VHH. The binding affinities of both LIFR-binding moieties are in a similar range, indicating that it is the fine epitope that discriminates between agonism and non-agonism. This is further supported from a structural perspective by the fact that VHH #3, which fails to induce agonism as bsAb, binds at the intersection between the D1 and D2 domains on LIFR, whereas VHH #8, inducing agonism as bsAb, targets a proximal epitope on LIFR (D2).

It was previously shown that multivalent targeting of receptor subunits substantially enhances cytokine-like agonism capacities (46). In general, sdAbs allow for multiple reformatting options as they do not feature a light chain 47, 48, 49. In this study, two different molecular design architectures were analyzed: tandem VHH arrangements and VHH domains engrafted onto the CH1 and CL of an IgG1 antibody (hidden IgG). Proliferation assays utilizing Ba/F3-gp130:LIFR cells and pSTAT3 experiments on HeLa cells naturally expressing both receptor subunits demonstrated that uncompromised binding to LIFR seems to be crucial for an effective LIF-like function. The orientation of the VHHs also appears to influence developability. Both molecular architectures resulted in candidates that demonstrated enhanced agonism compared to the parental bispecific molecule (1 + 1), giving evidence that valency engineering enables enhanced agonism capacities. Ultimately, this work adds LIF as a cytokine that can be mimicked using sdAb-derived bispecifics and supports that format architecture can have a tremendous impact on the agonistic capacities 50, 51, 52, 53, 54.

## Experimental procedures

### Camelid immunization

One female llama (*lama glama*), one female alpaca (*vicugna pacos*), and one male huarizo (*llama glama x vicugna pacos*) were immunized using His-tagged recombinant human LIFR (Sino Biological). The antigen was diluted with sterile deionized water according to the manufacturer’s recommendation to a final concentration of 0.9 mg/ml. The camelids were immunized subcutaneously with 0.3 mg total protein. Immunization was repeated three times on day 14, day 28 and day 35. On day 43, 100 ml whole blood was collected from each animal for PBMC isolation, RNA isolation and cDNA synthesis. After final blood collection, the animals remained alive. Animal procedures were performed at Preclinics GmbH, Germany, in accordance with local regulations and animal welfare protection laws. The study was approved by Niedersächsiches Landesamt für Verbraucherschutz und Lebensmittelsicherheit - Dezernat 33 – Tierschutzdienst.

### Yeast strains and media

The *Saccharomyces cerevisiae* strain EBY100 (MATa URA3-52 trp1 leu2Δ1 his3Δ200 pep4::HIS3 prb1Δ1.6R can1 GAL (pIU211:URA3)) (Thermo Fisher Scientific) was employed for yeast surface display. EBY100 were cultivated in yeast extract–peptone–dextrose (YPD) medium composed of 20 g/L peptone, 20 g/L dextrose, and 10 g/L yeast extract supplemented with 0.1 mg/ml penicillin–streptomycin (Gibco). After homologous recombination-based cloning, EBY100 cells harboring library plasmids (pDisp) were cultivated in medium composed of minimal synthetic defined (SD)-base (Takara Bio) and corresponding dropout mix (Takara Bio) containing all essential amino acids except for tryptophan (−Trp) for selection, supplemented with 5.4 g/L Na_2_HPO_4_ and 8.6 g/L NaH_2_PO_4_ × H_2_O. For induction of VHH gene expression, cells were transferred into galactose-containing SG dropout medium (−Trp), consisting of SG-base medium (Takara Bio) supplemented with 10% (w/v) polyethylene glycol 8000 (PEG 8000).

### Plasmids for yeast surface display and library generation

VHH yeast surface display libraries were generated utilizing homologous recombination-based gap repair cloning, described in detail by our group elsewhere (55). Briefly, VHH fragments amplified from the cDNA of immunized camelids are genetically fused to Aga2p after gap repair cloning in *BsaI-digested* display plasmid pDisp. The full-length VHH display on the yeast surface is detected *via* an HA epitope which is C-terminally linked to Aga2p on pDisp.

### Library sorting

EBY100 were cultured in SD medium (-Trp) overnight at 30 °C and 120 rpm until VHH surface expression was induced by transfer to SG medium (-Trp) at 10^7^ cells/ml and incubation for 48 h at 20 °C and 120 rpm. The YSD library was sorted over two consecutive rounds using 1 μM (rh) His-tagged LIFR ECD (Sino Biological, 10628-H08H). In the first round of sorting, antigen binding was detected *via* Penta His Alexa Fluor 647 Conjugate (QIAgen, 35370, RRID: AB_3083468, diluted 1:20), while anti-His mouse monoclonal detection antibody (SureLight Allophycocyanin, Abcam, ab72579, RRID: AB_1267597, diluted 1:20) was utilized for the second sorting round. To allow for two-dimensional sorting, full-length VHH surface presentation was simultaneously detected *via* a FITC-labeled rabbit polyclonal anti-HA antibody (Abcam, ab1208, RRID: AB_298835, diluted 1:20). For library sorting, a BD FACSAria Fusion cell sorter (BD Biosciences) device was used. For gate adjustment of the desired yeast cell population, untreated cells, cells incubated with detection reagents only and cells incubated with an unrelated antigen were analyzed. After two rounds of sorting, 192 clones of the enriched population were sequenced and a clonotyping strategy to cluster the VHHs based on their CDR3 region was applied as described elsewhere 46, 55.

### Protein expression, purification, and analytics

Proteins were expressed by transient transfection of 25 ml Expi293 cells according to the manufacturer’s instructions (Thermo Fisher Scientific) utilizing a 2:1 pTT5 plasmid ratio for SEEDbodies (2xAG:1xGA) and a 2:1:2 plasmid ratio for hidden IgG molecules (2xAG:1xGA:2xLC). After 5 to 7 days, protein containing supernatants were harvested by centrifugation and purified *via* MabSelect antibody purification chromatography resin (Cytiva). Following sterile filtration with Lyzer-CL GV 0.22 μm centrifugal devices (Merck Millipore), protein concentrations were measured using Nanodrop ND-1000 (Peqlab). Protein purities were evaluated *via* analytical SEC on a TSKgel UP-SW3000 column (2 μm, 4.6 × 300 mm, Tosoh Bioscience) using an Agilent HPLC 1260 Infinity system. 7.5 μg protein per sample were injected and a flow rate of 0.35 ml/min was applied employing 50 mM sodium phosphate, 0.4 M NaClO_4_ pH 6.3 as mobile phase.

### Generation of Ba/F3 cells with human LIF receptors

For the generation of Ba/F3 cells stably expressing gp130 and LIFR, retroviral transduction was performed using a virus-producing Phoenix Eco cell line (provided by DKFZ). Phoenix cells were seeded and transfected with pMOWS plasmids containing the human gp130 and human LIFR genes as well as resistance genes for puromycin or hygromycin B. For transfection, the transfection reagent TurboFect (Thermo Fisher Scientific) was used according to the manufacturer's instructions. Medium was exchanged to DMEM supplemented with 30% FBS and 1% penicillin-streptomycin (5000 U/ml) (P/S) 6 h after transfection. After a further 24 h of incubation, the supernatant was filtered through a filter with a pore size of 0.45 μm (Carl Roth). Subsequently, 1 × 10^5^ Ba/F3 cells were added to 250 μl of the virus-containing supernatant to a final volume of 300 μl, which additionally contained 8 μg/ml polybren (Merck KGaA). The cells were then centrifuged for 3 h at 1800 rpm at room temperature. Thereafter, cells were resuspended in 5 ml growth medium and cultured under standard conditions. The cells were selected with hygromycin after 24 h and with puromycin after 48 h.

### Analysis of Ba/F3-gp130:LIFR cell proliferation

Biologic activity of LIFR VHH-Fc binders and engineered molecules was assessed in a proliferation assay using Ba/F3-gp130:LIFR cells that proliferate in dependence of LIFR/gp130 binding. Before the experiment, cells were washed three times with PBS and resuspended in DMEM supplemented with 10% FBS and 1% P/S. 5 × 10^3^ cells/well were seeded into a 96-well plate and the VHH-Fc LIFR binders (100 nM) or the engineered LIF mimetics (1 nM) were added at a fixed concentration or in a 1:2 titration series starting at 1 nM to a final volume of 100 μl and incubated at 37 °C for 72 h. As a positive control, Ba/F3-gp130:LIFR cells were stimulated with 2 μl/ml cell supernatant from a stable CHO-K1 cell clone that secretes hyper-IL-6, which is an IL-6/IL-6R fusion protein (stock solution approx. 10 μg/ml, determined by ELISA, designated “Hy”). Unstimulated cells were included as a negative control. To detect proliferation, 20 μl CellTiter-Blue reagent (Promega) were added to each well. Fluorescence was determined *via* Tecan Infinite M200 PRO Reader (Tecan) at an excitation wavelength of 560 nm and an emission of 590 nm every 20 min over 2 h. For evaluation, the background signal (time point 0) was subtracted from the final fluorescence intensity. The data are presented as relative proliferation, normalized to the respective positive and negative controls. Standard deviation is indicated by error bars. The measurements were conducted in three biological replicates, each with three technical replicates.

### Biolayer interferometry

All Biolayer interferometry (BLI) measurements were performed using the Octet RED96 system (ForteBio, Pall Life Science) at 25 °C and 1000 rpm. The data were analyzed and fitted with ForteBio data analysis software 12.2, applying Savitzky-Golay filtering and a 1:1 binding model as needed. All steps were performed in kinetics buffer (KB; PBS + 0.1% Tween-20 and 1% bovine serum albumin, BSA).

For determination of binding affinities (KD), molecules were immobilized on anti-human Fc capture (AHC) biosensors at 5 μg/ml for 180 s. After immobilization, biosensors were rinsed for 45 s in KB prior to association. Binding to (rh) His-tagged LIFR ECD (Sino Biological) or (rh) His-tagged gp130 ECD (Acro Biosystems) was measured during a 300 s association step, followed by a dissociation step in KB for 300 s (engineered LIF mimetics) or 600 s (monospecific VHH-Fc LIFR binders). For the engineered LIF mimetics, (rh) LIFR-His was applied in a 1:2 dilution series ranging from 250 nM to 31.25 nM, and (rh) gp130-His was used in a 1:2 dilution series ranging from 62.5 nM to 7.8 nM. The KD of LIFR binder #8 was determined from a 1:2 (rh) LIFR-His dilution series ranging from 125 nM to 7.8 nM. Binding kinetics of other monospecific VHH-Fc LIFR binders were measured at the three concentrations (200 nM, 50 nM and 12.5 nM).

To analyze competitive binding of VHH-Fc LIFR binders and (rh) LIF-Fc (Sino Biological) to (rh) LIFR-His (Sino Biological) or VHH-Fc LIFR binder #8 and other VHH-Fc LIFR binders, (rh) LIFR-His was loaded at 5 μg/ml onto HIS1K biosensors for 180 s, followed by 45 s sensor rinsing in KB. The first association step was performed for 300 s either in 125 nM of VHH-Fc LIFR binders or in VHH-Fc LIFR binder #8, followed by a second association step for 300 s in 125 nM of (rh) LIF-Fc or the remaining VHH-Fc LIFR binders.

Simultaneous binding of the engineered LIF mimetics was evaluated by loading of AHC biosensors with the molecules at 5 μg/ml, rinsing the sensor in KB for 45 s and performing two consecutive association steps for 300 s each. The first association was performed in 125 nM (rh) LIFR-His (Sino Biological), and for the second association step, 125 nM (rh) gp130-His (Acro Biosystems) spiked with 75 nM (rh) LIFR-His was used.

### Cryo-EM data collection, processing, and model building

LIFR-VHH complexes were prepared by mixing VHH in a 1:1 M ratio and incubated for an hour at room temperature. Glow-discharged Quantifoil R 1.2/1.3300 mesh gold grids (Electron Microscopy Sciences) were loaded into a Vitrobot Mark IV system with the following settings: 4 °C chamber, 100% humidity, blot force 1, wait time 5 s, and variable blot times (3–5.5 s). 3 μL of complex sample was applied before the grids were blotted and plunge-frozen in liquid ethane. The concentration of the complex was 0.5 mg/ml.

Samples were imaged at 190,000× nominal magnification using a Falcon 4i camera on a Glacios microscope at 200 kV. Automated image collection was performed using EPU from ThermoFisher. Images were aligned, dose-weighted, and Contrast Transfer Function (CTF)-corrected in the CryoSPARC Live (56) software platform, with automated image collection also performed using Smart EPU software (ThermoFisher).

Data processing was carried out in CryoSPARC v4.7.1. Blob particle picking was performed on all micrographs with a minimum particle diameter of 130 Å and a maximum of 250 Å. Particles were extracted and used to perform 2D classification; representative classes were utilized to conduct new template-based particle picking to improve detection of rare views.

High-quality 2D class averages from the template-picked particles were used as input for Topaz training, enabling the selection of bound LIF complexes and the generation of an initial 3D reference *via ab initio* reconstruction. This initial model was further refined through multiple rounds of heterogeneous refinements and 3D classifications, yielding a single high-quality class that was subsequently refined using homogenous and local refinements.

To model the complexes, we predicted the nanobody structures using AlphaFold3 (57) and used the available PDB structures of LIF and gp130 (58) as templates for the antigen structures. As AlphaFold2/3 and other co-folding tools struggled with predicting the nanobody binding sites, we docked the initial individual models into the cryoEM density maps in UCSF ChimeraX (59) and refined the structures within the density using Rosetta relax 60, 61 and cryo-EM density-guided simulations implemented in GROMACS (62). The structure model was built iteratively with COOT (63).

### Cell culture

HeLa cells (DSMZ) were cultured in Dulbecco's Modified Eagle's Medium (DMEM) (Gibco) supplemented with 10% heat-inactivated FBS (Gibco). Cell count was maintained between 0.1 to 1.5 × 10^6^ cells/ml by splitting the cells three times a week. For passaging, adherent HeLa cells were washed once with PBS and cells were detached from the cell culture flask using trypsin. TF-1 cells (ATCC) were cultured in RPMI 1640 Medium with GlutaMAX Supplement (Gibco) supplemented with 10% FBS (Sigma-Aldrich), 1 mM Sodium Pyruvate (Gibco) and 2.5 g/L Glucose (Gibco). Cells were passaged 2 to 3 times a week below 0.8 × 10^6^ cells/ml with 2 ng/ml (rh) GM-CSF (R&D) freshly added to the culture flask. All cells were cultivated at 37 °C and 5% CO_2_.

### pSTAT3 detection by flow cytometry

For detection of STAT3 signaling induced by engineered LIF mimetics, TF-1 or HeLa cells were washed with PBS and resuspended in respective media at 1.0 × 10^6^ vc/ml (TF-1) or 1.7 × 10^6^ vc/ml (HeLa). 5 × 10^4^ cells/well (TF-1) or 8.5 × 10^4^ cells/well (HeLa) were transferred in a sterile, round-bottom, 96-well plate (Thermo Fisher Scientific). The cells showed a minimum viability of 94%. (Rh) LIF cytokine (Acro Biosystems) and mimetic samples were prepared in the respective media and titrated as 1:5 serial dilutions to assess dose dependence. Cells were incubated with the samples for 40 min at 37 °C and 5% CO_2_ under humidified conditions. Afterwards, cells were fixed by applying 190 μl/well of Lyse/Fixation Buffer (BD Biosciences) and incubating for 10 min at 37 °C and 350 rpm on a plate shaker. For permeabilization, cells were washed twice with washing buffer (PBS supplemented with 0.5% BSA) and then resuspended in 150 μl pre-cooled methanol (−20 °C). After 30 min of incubation at 4 °C, cells were washed twice and stained for intracellular pSTAT3 (pSTAT3 AlexaFluor488, BD, 557814). As an isotype control, Mouse IgG2a, κ, AF488 (BD, 558055) was used. All antibodies were diluted in washing buffer. After two final washes, cells were resuspended in 20 μl (TF-1) or 25 μl (HeLa) washing buffer and analyzed using flow cytometry (IQue3, Sartorius). Data were analyzed with iQue Forecyt Software (v8.0) and plotted in GraphPad Prism (v10). The measurements were performed in four (TF-1) or six (HeLa) biological replicates. Error bars show standard error of the mean.

## Data availability

The cryo-EM density maps for LIFR in complex with VHH #8 and VHH #3, as well as gp130 in complex with the anti-gp130 VHH, have been deposited in the Electron Microscopy Data Bank (EMDB) under accession codes EMD-77922, EMD-77923, and EMD-77938, respectively.

As described in the Results section, the cryo-EM maps did not reach sufficient resolution (∼4–5 Å) for confident atomic model building. The density-informed structural models presented herein integrate experimental cryo-EM density with structure prediction and serve for topology visualization only. For the full bsAb:LIFR:gp130 complex, a 3D reconstruction of sufficient quality for map deposition could not be obtained; the 2D class averages shown in Figure 4*D* represent the available structural data for this assembly.

## Supporting information

This article contains supporting information.

## Conflict of interest

The authors declare the following financial interests/personal relationships which may be considered as potential competing interests: JH, BL, LU, EG, DE, LP, AE and SZ are affiliated with Merck Healthcare KGaA. Besides, all authors declare that they have no known competing financial interest or personal relationships that could have appeared to influence the work reported in this paper.

## References

[1] Jones S.A., Jenkins B.J. (2018). Recent insights into targeting the IL-6 cytokine family in inflammatory diseases and cancer. Nat. Rev. Immunol..

[2] Kang S., Narazaki M., Metwally H., Kishimoto T. (2020). Historical overview of the interleukin-6 family cytokine. J. Exp. Med..

[3] Scheller J., Ettich J., Wittich C., Pudewell S., Floss D.M., Rafii P. (2024). Exploring the landscape of synthetic IL -6-type cytokines. FEBS J..

[4] Nicola N.A., Babon J.J. (2015). Leukemia inhibitory factor (LIF). Cytokine Growth Factor Rev..

[5] Wang J., Chang C.Y., Yang X., Zhou F., Liu J., Feng Z. (2023). Leukemia inhibitory factor, a double-edged sword with therapeutic implications in human diseases. Mol. Ther..

[6] Jorgensen M.M., De La Puente P. (2022). Leukemia inhibitory factor: an important cytokine in pathologies and cancer. Biomolecules.

[7] Gearing D.P., Gough N.M., King J.A., Hilton D.J., Nicola N.A., Simpson R.J. (1987). Molecular cloning and expression of cDNA encoding a murine myeloid leukaemia inhibitory factor (LIF). EMBO J..

[8] Chen J.R., Cheng J.G., Shatzer T., Sewell L., Hernandez L., Stewart C.L. (2000). Leukemia inhibitory factor can substitute for nidatory estrogen and is essential to inducing a receptive uterus for implantation but is not essential for subsequent Embryogenesis^1^. Endocrinology.

[9] Stewart C.L., Kaspar P., Brunet L.J., Bhatt H., Gadi I., Köntgen F. (1992). Blastocyst implantation depends on maternal expression of leukaemia inhibitory factor. Nature.

[10] Song H., Lim H., Das S.K., Paria B.C., Dey S.K. (2000). Dysregulation of EGF family of growth factors and COX-2 in the uterus during the preattachment and attachment reactions of the blastocyst with the luminal epithelium correlates with implantation failure in LIF- deficient mice. Mol. Endocrinol..

[11] Williams R.L., Hilton D.J., Pease S., Willson T.A., Stewart C.L., Gearing D.P. (1988). Myeloid leukaemia inhibitory factor maintains the developmental potential of embryonic stem cells. Nature.

[12] Yoshida T., Satoh M., Nakagaito Y., Kuno H., Takeuchi M. (1993). Cytokines affecting survival and differentiation of an astrocyte progenitor cell line. Develop. Brain Res..

[13] Mayer M., Bhakoo K., Noble M. (1994). Ciliary neurotrophic factor and leukemia inhibitory factor promote the generation, maturation and survival of oligodendrocytes in vitro. Development.

[14] Fischer R., Wajant H., Kontermann R., Pfizenmaier K., Maier O. (2014). Astrocyte-specific activation of TNFR2 promotes oligodendrocyte maturation by secretion of leukemia inhibitory factor. Glia.

[15] Davis S.M., Pennypacker K.R. (2018). The role of the leukemia inhibitory factor receptor in neuroprotective signaling. Pharmacol. Ther..

[16] Duluc D., Delneste Y., Tan F., Moles M.P., Grimaud L., Lenoir J. (2007). Tumor-associated leukemia inhibitory factor and IL-6 skew monocyte differentiation into tumor-associated macrophage-like cells. Blood.

[17] Yaftiyan A., Eskandarian M., Jahangiri A.H., Kazemi Sefat N.A., Moazzeni S.M. (2019). Leukemia inhibitory factor (LIF) modulates the development of dendritic cells in a dual manner. Immunopharmacol. Immunotoxicol.

[18] Gao W., Thompson L., Zhou Q., Putheti P., Fahmy T.M., Strom T.B. (2009). Treg versus Th17 lymphocyte lineages are cross-regulated by LIF versus IL-6. Cell Cycle.

[19] Christianson J., Oxford J.T., Jorcyk C.L. (2021). Emerging perspectives on leukemia inhibitory factor and its receptor in cancer. Front. Oncol..

[20] Halder S., Parte S., Kshirsagar P., Muniyan S., Nair H.B., Batra S.K. (2022). The Pleiotropic role, functions and targeted therapies of LIF/LIFR axis in cancer: old spectacles with new insights. Biochim. Biophys. Acta (Bba) - Rev. Cancer.

[21] Viswanadhapalli S., Dileep K.V., Zhang K.Y.J., Nair H.B., Vadlamudi R.K. (2022). Targeting LIF/LIFR signaling in cancer. Genes Dis..

[22] Boulanger M.J., Bankovich A.J., Kortemme T., Baker D., Garcia K.C. (2003). Convergent mechanisms for recognition of divergent cytokines by the shared Signaling receptor gp130. Mol. Cell.

[23] Huyton T., Zhang J.G., Luo C.S., Lou M.Z., Hilton D.J., Nicola N.A. (2007). An unusual cytokine:Ig-domain interaction revealed in the crystal structure of leukemia inhibitory factor (LIF) in complex with the LIF receptor. Proc. Natl. Acad. Sci. U. S. A..

[24] Skiniotis G., Lupardus P.J., Martick M., Walz T., Garcia K.C. (2008). Structural Organization of a full-length gp130/LIF-R cytokine receptor transmembrane complex. Mol. Cell.

[25] Valldorf B., Hinz S.C., Russo G., Pekar L., Mohr L., Klemm J. (2022). Antibody display technologies: selecting the cream of the crop. Biol. Chem..

[26] Pudewell S., Heuser J., Dorogobed I., Lipinski B., Tran T.H.H., Metzenmacher P. (2025). Targeting the major pro-inflammatory interleukin-6-type cytokine receptor gp130 by antagonistic single domain antibodies. Front. Immunol..

[27] Labrijn A.F., Meesters J.I., de Goeij B.E.C.G., van den Bremer E.T.J., Neijssen J., van Kampen M.D. (2013). Efficient generation of stable bispecific IgG1 by controlled Fab-arm exchange. Proc. Natl. Acad. Sci. U. S. A..

[28] Yanakieva D., Pekar L., Evers A., Fleischer M., Keller S., Mueller-Pompalla D. (2022). Beyond bispecificity: controlled Fab arm exchange for the generation of antibodies with multiple specificities. mAbs.

[29] Barron N., Dickgiesser S., Fleischer M., Bachmann A.N., Klewinghaus D., Hannewald J. (2024). A generic approach for miniaturized unbiased high-throughput screens of bispecific antibodies and biparatopic antibody–drug conjugates. IJMS.

[30] Tang Y., Tian X. (2013). (Cindy). JAK-STAT3 and somatic cell reprogramming. JAK-STAT.

[31] Chollangi S., Mather T., Rodgers K.K., Ash J.D. (2012). A unique loop structure in Oncostatin M determines binding affinity toward Oncostatin M receptor and leukemia inhibitory factor receptor. J. Biol. Chem..

[32] Zhou Y., Stevis P.E., Cao J., Ehrlich G., Jones J., Rafique A. (2024). Structures of complete extracellular assemblies of type I and type II Oncostatin M receptor complexes. Nat. Commun..

[33] Pekar L., Busch M., Valldorf B., Hinz S.C., Toleikis L., Krah S. (2020). Biophysical and biochemical characterization of a VHH-based IgG-like bi- and trispecific antibody platform. mAbs.

[34] Davis J.H., Aperlo C., Li Y., Kurosawa E., Lan Y., Lo K.M. (2010). SEEDbodies: fusion proteins based on strand-exchange engineered domain (SEED) CH3 heterodimers in an Fc analogue platform for asymmetric binders or immunofusions and bispecific antibodies. Protein Eng. Des. Sel..

[35] Thoma B., Bird T.A., Friend D.J., Gearing D.P., Dower S.K. (1994). Oncostatin M and leukemia inhibitory factor trigger overlapping and different signals through partially shared receptor complexes. J. Biol. Chem..

[36] Ikezono T., Wu T., Yao X.L., Levine S., Logun C., Angus C.W. (1997). Leukemia inhibitory factor induces the 85-kDa cytosolic phospholipase A2 gene expression in cultured human bronchial epithelial cells. Biochim. Biophys. Acta (Bba) - Mol. Cell Res..

[37] Gao B., Liu H., Zhu M., Zhang S., Wang M., Ruan Y. (2025). Molecular dynamics simulations reveal key roles of the LIF receptor in the assembly of human LIF signaling complex. Comput. Struct. Biotechnol. J..

[38] Saxton R.A., Glassman C.R., Garcia K.C. (2023). Emerging principles of cytokine pharmacology and therapeutics. Nat. Rev. Drug Discov..

[39] Berraondo P., Sanmamed M.F., Ochoa M.C., Etxeberria I., Aznar M.A., Pérez-Gracia J.L. (2019). Cytokines in clinical cancer immunotherapy. Br. J. Cancer.

[40] Xue D., Hsu E., Fu Y.-X., Peng H. (2021). Next-generation cytokines for cancer immunotherapy. Antibody Ther..

[41] Pires I.S., Hammond P.T., Irvine D.J. (2021). Engineering strategies for immunomodulatory cytokine therapies: challenges and clinical progress. Adv. Ther..

[42] Pinho V., Fernandes M., Da Costa A., Machado R., Gomes A.C. (2020). Leukemia inhibitory factor: recent advances and implications in biotechnology. Cytokine Growth Factor Rev..

[43] Warnke C., Hermanrud C., Lundkvist M., Fogdell-Hahn A. (2012). Anti-drug antibodies. Drugs Ther. Stud..

[44] Moraga I., Wernig G., Wilmes S., Gryshkova V., Richter C.P., Hong W.J. (2015). Tuning cytokine receptor signaling by Re-orienting dimer geometry with surrogate ligands. Cell.

[45] Mohan K., Ueda G., Kim A.R., Jude K.M., Fallas J.A., Guo Y. (2019). Topological control of cytokine receptor signaling induces differential effects in hematopoiesis. Science.

[46] Lipinski B., Unmuth L., Arras P., Becker S., Bauer C., Toleikis L. (2023). Generation and engineering of potent single domain antibody-based bispecific IL-18 mimetics resistant to IL-18BP decoy receptor inhibition. mAbs.

[47] Chanier T., Chames P. (2019). Nanobody engineering: toward next generation immunotherapies and immunoimaging of cancer. Antibodies.

[48] Lipinski B., Arras P., Pekar L., Klewinghaus D., Boje A.S., Krah S. (2023). NKp46 -specific single domain antibodies enable facile engineering of various potent NK cell engager formats. Protein Sci..

[49] Evers A., Guarnera E., Pekar L., Zielonka S. (2025). From discovery to the clinic: structural insights, engineering options, clinical, and ‘next wave’ applications of camelid-derived single-domain antibodies. mAbs.

[50] Pekar L., Krah S., Zielonka S. (2024). Taming the beast: engineering strategies and biomedical potential of antibody-based cytokine mimetics. Expert Opin. Biol. Ther..

[51] Lipinski B., Unmuth L., Arras P., Endruszeit R., Becker S., Rödel J.M. (2025). Taming interleukin-12: engineering of bispecific antibody-based IL -12 mimetics with biased agonism capacities. Protein Sci..

[52] Lipinski B., Pekar L., Evers A., Unmuth L., Guarnera E., Zielonka S. (2026). Multifunctional antibody-derived IL-12 mimetics: prospects of future clinical development. Expert Opin. Investig. Drugs.

[53] Unmuth L., Lipinski B., Hoerr A., Harwardt J., Guarnera E., Szczepek M. (2026). Antigen-directed single domain antibody-based TNFR1 agonists elicit preferential killing of HER2-overexpressing cancer cells. iScience.

[54] Lipinski B., Unmuth L., Tran T.H.H., Harwood J., Arras P., Becker S. (2026). Antibody framework engineering enables augmented cytokine receptor signaling capacities of bispecific single domain antibody-based IL-21 mimetics. mAbs.

[55] Roth L., Krah S., Klemm J., Günther R., Toleikis L., Busch M., Zielonka S., Krah S. (2020).

[56] Punjani A., Rubinstein J.L., Fleet D.J., Brubaker M.A. (2017). cryoSPARC: algorithms for rapid unsupervised cryo-EM structure determination. Nat. Methods.

[57] Abramson J., Adler J., Dunger J., Evans R., Green T., Pritzel A. (2024). Accurate structure prediction of biomolecular interactions with AlphaFold 3. Nature.

[58] Zhou Y., Stevis P.E., Cao J., Saotome K., Wu J., Glatman Zaretsky A. (2023). Structural insights into the assembly of gp130 family cytokine signaling complexes. Sci. Adv..

[59] Pettersen E.F., Goddard T.D., Huang C.C., Meng E.C., Couch G.S., Croll T.I. (2021). UCSF ChimeraX : structure visualization for researchers, educators, and developers. Protein Sci..

[60] Nivón L.G., Moretti R., Baker D. (2013). A pareto-optimal refinement method for protein design scaffolds. PLoS ONE.

[61] Conway P., Tyka M.D., DiMaio F., Konerding D.E., Baker D. (2014). Relaxation of backbone bond geometry improves protein energy landscape modeling. Protein Sci..

[62] Shugaeva T., Haloi N., Cowgill J., Villa A. (2024). GROMACS Tutorial: Density Fit Simulations..

[63] Emsley P., Lohkamp B., Scott W.G., Cowtan K. (2010). Features and development of *Coot*. Acta Crystallogr. D Biol. Crystallogr..

[64] Meng E.C., Goddard T.D., Pettersen E.F., Couch G.S., Pearson Z.J., Morris J.H. (2023). UCSF ChimeraX : tools for structure building and analysis. Protein Sci..

