# Supplementary material for: LIF and let die: Generation, mechanistical, and structural characterization of bispecific antibodies triggering leukemia inhibitory factor (LIF) receptor activation independent of the cytokine LIF
Source: J Biol Chem. 2026 Oct 9;302(11):113634. doi: 10.1016/j.jbc.2026.113634 (PMC13753589; doi:10.1016/j.jbc.2026.113634)
Supplement: Supplementary file 1 [file mmc1.docx]

**Supplementary Figures**

**Figure S1: Binding characterization of VHH-Fc LIFR binders determined *via* BLI measurements.** (A) Binding kinetics of reformatted VHH-Fc LIFR targeting molecules identified by YSD. The sdAbs were immobilized on AHC biosensors. (Rh) LIFR was associated at three concentrations (200 nM, 50 nM, 12.5 nM) for 300 s, followed by dissociation for 600 s. The data was fitted using a 1:1 binding model after Savitzky-Golay filtering (red lines). Molecule #8 was loaded on AHC biosensors and consecutively associated with increasing (rh) LIFR concentrations ranging from 125 nM to 7.81 nM for 300s, followed by dissociation for 600s. The data was fitted using a 1:1 binding model after Savitzky-Golay filtering (blue lines). (B) Sequence alignment of LIFR binders #4 and #8. CDRs are highlighted. Sequence identity is shown in green. Alignment conducted with MUSCLE alignment tool using Geneious Prime 2025.0.2.

**Figure S2: Proliferation assay on Ba/F3-gp130:LIFR cells.** Molecule 8 and LIF were titrated to the cells in a concentration range between 0.061 pM and 1nM. Hyper-IL-6 (Hy) was used as positive control. Every value is measured in triplicates.

**Figure S3: BLI competition assay of 14 VHH-Fc LIFR binders and (rh) LIF-Fc.** (Rh) LIFR was loaded onto HIS1K biosensors. The first association was performed with 125 nM of the corresponding VHH-Fc molecule (#1-14) for 300 s and the second association was conducted with 125 nM (rh) LIF-Fc for 300 s. Values are normalized to the start of the second association.

**Figure S4: Analytical size exclusion chromatography (SEC) profiles.** SEC-HPLC profiles and corresponding target monomer peaks post protein A purification of different formats are shown. Absorbance at 214 nm was utilized for purity determination.

**Figure S5: BLI-assisted simultaneous antigen binding measurement of bispecific engineered LIF mimetics.** LIFR and gp130 targeting molecules were loaded onto AHC biosensors and sequentially associated to 125 nM (rh) LIFR and 125 nM (rh) gp130 for 300 s each.

**Figure S6: Binding kinetics of engineered LIF mimetics measured *via* BLI.** (A) Molecules were immobilized on AHC biosensors. (Rh) LIFR was associated at four concentrations (250 nM, 125 nM, 62.5 nM, 31.3 nM) for 300 s, followed by dissociation for 300 s. (B) Molecules were immobilized on AHC biosensors. (Rh) gp130 was associated at four concentrations (62.5 nM, 31.3 nM, 15.6 nM, 7.8 nM) for 300 s, followed by dissociation for 300 s. The data was fitted using a 1:1 binding model after Savitzky-Golay filtering (colored lines).

**Figure S7: Proliferation assay of Ba/F3-gp130:LIFR cells.** Engineered molecules were titrated to the cells in a 1:2 titration series starting at 1 nM. Hyper-IL-6 (Hy) was used as a positive control. Every value is measured in triplicates and all assays were performed in three independent experiments (n=3).

**Figure S8: Intracellular staining of pSTAT3^+^ TF-1 cells.** Phosphorylation of STAT3 was stained after 40 min incubation with (rh) LIF or LIFR agonists at different concentrations. Mean values ± SEM of four independent measurements are depicted. The dose response curves were fitted using a non-linear four parameter fit in GraphPad Prism.
